# Basal metabolic rate mediates the causal relationship between gut microbiota and osteoarthritis: a two-step bidirectional Mendelian randomization study

**DOI:** 10.3389/fmicb.2024.1371679

**Published:** 2024-10-01

**Authors:** Jiachen Li, Jianhui Liang, Yang Liu, Weichao Sun, Wei Sun

**Affiliations:** ^1^Department of Orthopedics, Shenzhen Second People's Hospital/First Affiliated Hospital of Shenzhen University, Shenzhen, Guangdong, China; ^2^Shantou University Medical College, Shantou, China; ^3^The Central Laboratory, Shenzhen Second People's Hospital/First Affiliated Hospital of Shenzhen University, Shenzhen, Guangdong, China

**Keywords:** basal metabolic rate, gut microbiota, mediation, Mendelian randomization, osteoarthritis

## Abstract

**Background:**

The relationship between gut microbiota and osteoarthritis (OA) occurrence remains unclear. Existing research needs to clearly understand how basal metabolic rate (BMR) regulates this relationship. Therefore, using a two-step bidirectional Mendelian Randomization approach, our study aims to investigate whether BMR levels mediate the causal relationship between gut microbiota and OA.

**Methods:**

In this study, we examined publicly available summary statistics from Genome-Wide Association Studies (GWAS) to determine the correlation between gut microbiota and OA. The analysis included one primary dataset and two secondary datasets. Initially, a two-step, two-sample, and reverse MR analysis was performed to identify the causal relationship between gut microbiota and OA. Subsequently, a two-step MR analysis revealed that the relationship between microbiota and OA is mediated by BMR. Sensitivity analyses confirmed the robustness of the study results.

**Results:**

In our analysis of the primary dataset, we discovered a positive correlation between three taxa and the outcome of OA, and eight taxa exhibited a negative correlation with the OA outcome. Through comparisons with the secondary dataset and multiple testing corrections, we found a negative association between the class *Actinobacteria* (OR=0.992886277, *p*-value = 0.003) and the likelihood of OA occurrence. Notably, knee osteoarthritis (KOA) and hip osteoarthritis (HOA) had a strong negative correlation (OR = 0.927237553/0.892581219). Our analysis suggests that BMR significantly mediates the causal pathway from *Actinobacteria* to OA, with a mediated effect of 2.59%. Additionally, BMR mediates 3.98% of the impact in the path from the order *Bifidobacteriales* and the family *Bifidobacteriaceae* to OA. Besides these findings, our reverse analysis did not indicate any significant effect of OA on gut microbiota or BMR.

**Conclusion:**

Our research results indicate that an increase in the abundance of specific gut microbial taxa is associated with a reduced incidence of OA, and BMR levels mediate this causal relationship. Further large-scale randomized controlled trials are necessary to validate the causal impact of gut microbiota on the risk of OA. This study provides new insights into the potential prevention of OA by modulating the gut microbiota.

## 1 Introduction

Osteoarthritis (OA) is a degenerative disease that affects the entire joint. The typical features of OA included synovial inflammation, cartilage loss, joint osteophyte formation, meniscal injury, and the degeneration of tendons and ligaments (Gu et al., 2018; Wei and Bao, 2022). OA is a condition that causes disability, and its occurrence and prevalence are increasing among the general population. OA is primarily associated with aging. Its prevalence is projected to steadily rise and is expected to become the leading cause of disability in the general population by the year 2030 (Thomas et al., 2014). OA predominantly affects weight-bearing joints in the human body. A recent estimate indicates approximately 300 million cases of hip and knee OA (GBD 2015 Disease and Injury Incidence and Prevalence Collaborators, 2016). As a prevalent musculoskeletal disease, it imposes a substantial healthcare burden, impacting individuals and placing strain on healthcare systems (Giorgino et al., 2023). Due to its complex and unexplained pathogenesis, joint replacement is the only option in the end stage, causing pain to patients and a heavy burden on society. Therefore, researching early intervention strategies for osteoarthritis remains crucial in alleviating the overall health burden on society.

The gut microbiota actively participates in various physiological functions, including metabolism, immune and neural functions, metabolic stability maintenance, immune system development, resistance to infections, and the production of certain neurotransmitters (Adak and Khan, 2019). With the continuous advancement of high-throughput sequencing technologies and platforms, there is a growing body of evidence indicating that gut microbiota has a significant impact on skeletal metabolism. Certain microbial communities in the intestines, for instance, can produce short-chain fatty acids like butyrate, propionate, and acetate. These fatty acids are crucial for maintaining skeletal health (Blaak et al., 2020). Butyrate has been discovered to boost osteoblasts’ functionality and hinder osteoclasts’ activity, thus supporting the overall well-being of bones (Medawar et al., 2021). Due to the susceptibility of the gut microbiota balance to various factors, disturbances may lead to conditions such as obesity, diabetes, metabolic disorders, and even cancer in individuals (Xu et al., 2020).

The basal metabolic effect constitutes approximately 70% of daily energy expenditure (Levine, 2005), and its efficiency is referred to as the basal metabolic rate (BMR). BMR plays a vital role in upholding normal structure and function, and any discrepancy between metabolic requirements and BMR is strongly associated with clinical pathologies. In previous investigations, common risk factors for OA have been identified, including obesity (Johnson and Hunter, 2014; Allen and Golightly, 2015; Neogi and Zhang, 2013; Lane et al., 2017), hyperlipidemia, and other factors that reflect features associated with metabolic syndrome and manifestations of metabolic aberrations. Metabolism is a fundamental aspect of life, in which the human body utilizes nutrients obtained from dietary intake for energy metabolism to sustain physiological functions. According to previous studies (Wei et al., 2022), the gut microbiota may play a role in the onset of OA by affecting organismal metabolism or interactions. The basal metabolic rate may be an intermediary factor in mediating the occurrence of osteoarthritis. Therefore, it is imperative to determine the potential interplay between BMR levels and gut microbiota in the development of OA.

Mendelian Randomization (MR) analysis utilizes genetic variations as instrumental variables for exposure to establish causality between exposure and outcomes (Greenland, 2018). Since common confounding factors do not influence the random distribution of allele genes, causal relationships are commonly regarded as dependable (Burgess et al., 2013). However, no previous MR studies have been identified that investigate the correlation between gut microbiota, OA, and their connections with BMR. Hence, we conducted a two-step, two-sample bidirectional MR analysis based on summary statistics from Genome-Wide Association Studies (GWAS) to assess the causal relationships between gut microbiota, BMR, and OA.

## 2 Materials and methods

### 2.1 Study design

**Figure 1:**
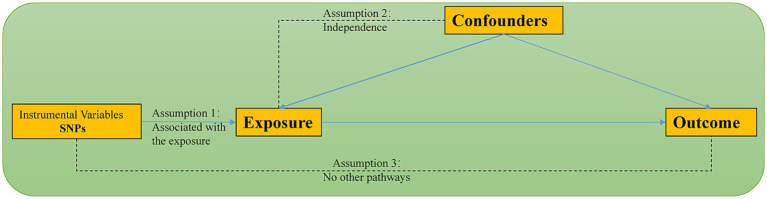
The schematic representation of the basic methodology used in MR analysis.

This research employed a two-sample, two-step bidirectional Mendelian Randomization (MR) design based on summary data from Genome-Wide Association Studies (GWAS). The study followed the most recent recommendations for conducting MR analysis as outlined in the STROBE-MR guidelines and was based on three fundamental assumptions: (1) Instrumental variables are closely associated with gut microbiota. (2) Any potential confounding factors do not influence the chosen instrumental variables. (3) The chosen instrumental variables are unrelated to the outcome but related to the exposure. Additionally, meeting other assumptions requires the absence of statistical interactions (Skrivankova et al., 2021). Figure 1 illustrates the schematic representation of the methodology used in MR analysis.

**Figure 2:**
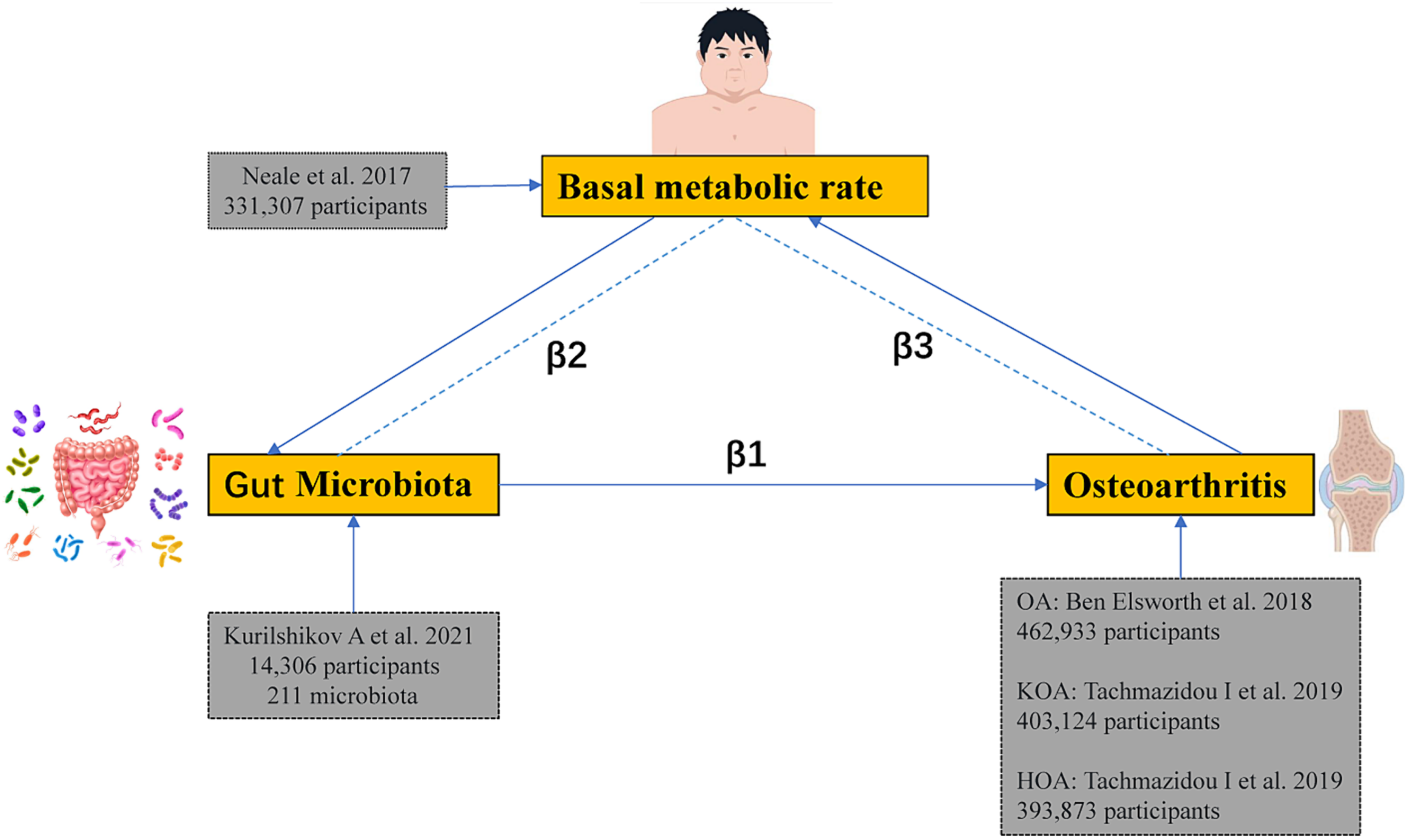
The schematic representation of the study design in this article.

Firstly, an MR analysis was conducted to determine the association between gut microbiota and OA to obtain the total effect size β1. We then conducted another MR analysis on the gut microbiota and the mediator to determine the effect size β2. Lastly, an additional MR analysis was conducted to investigate the link between BMR and OA, resulting in the effect size β3. Additionally, a reverse Mendelian Randomization study was conducted to eliminate the possibility of bidirectional causation. The study utilized GWAS summary-level data, for which appropriate informed consent and ethical approvals were obtained from the original studies. The schematic representation of the study design is illustrated in Figure 2.

### 2.2 Data source

The gut microbiota data used in this study originated from the MiBioGen Consortium (Kurilshikov et al., 2021). The consortium curated and analyzed genetic information of whole genomes and 16S fecal microbiome data from a total of 18,340 individuals across 24 cohorts. This included data from 14,306 individuals with European ancestry who participated in 18 different cohorts. The consortium adjusted for technical covariates, such as age, gender, genetic principal components, and fecal DNA extraction methods, as well as 16S structural domains, aiming to reduce heterogeneity across cohorts.

The GWAS summary data for the European BMR were sourced from the IEU Open GWAS database.^1^ This database represents an analysis of the UK Biobank (Rusk, 2018) phenotype survey results conducted by the Medical Research Council-Integrative Epidemiology Unit (MRC-IEU) consortium (Elsworth and et.al, 2020; Hemani et al., 2018).

The outcome data analyzed in this study were obtained from the Medical Research Council Integrative Epidemiology Unit Open GWAS.^2^ The primary outcome data originated from the OA dataset, which included 462,933 European individuals. This dataset comprised 424,461 healthy controls and 38,472 OA patients, with 9,851,867 SNPs. Site-specific secondary outcome data were derived from two datasets of the same GWAS, representing a meta-analysis of the entire OA genome using UK Biobank and ArcGEN resources (Tachmazidou et al., 2019). The KOA dataset included 378,169 healthy controls and 24,955 knee OA patients. The HOA dataset included 378,169 healthy controls and 15,704 hip OA patients. Table 1 summarizes the GWAS data used in this study.

**Table 1:** A comprehensive summary of the GWAS data sources and information utilized.

Variable	GWAS ID/PMID	Data sources	Population	Sample size	Web resource
Exposure					
Gut microbiota	PMID: 33462485	MiBioGen consortium	European	18,340	https://mibiogen.gcc.rug.nl/
Mediation					
Basal metabolic rate	ukb-a-268	Neale Lab	European	331,307	https://gwas.mrcieu.ac.uk/datasets/ukb-a-268/
Outcome					
Osteoarthritis	ukb-b-14486	MRC-IEU	European	462,933	https://gwas.mrcieu.ac.uk/datasets/ukb-b-14486/
Knee osteoarthritis	ebi-a-GCST007090	NA	European	403,124	https://gwas.mrcieu.ac.uk/datasets/ebi-a-GCST007090/
Hip osteoarthritis	ebi-a-GCST007091	NA	European	393,873	https://gwas.mrcieu.ac.uk/datasets/ebi-a-GCST007091/

### 2.3 SNP selection

We used genetic variation as instrumental variables (IVs). To satisfy assumption 2, we ensured that the selected Single Nucleotide Polymorphisms (SNPs) were strongly associated with gut microbiota, BMR, and OA, respectively. We initially selected SNPs from the MiBioGen data for gut microbiota that exhibited genome-wide significant associations (*p* < 5×10-08) with the exposure. In cases where the number of IVs was limited, the significance threshold was relaxed to 1×10-05. Subsequently, based on prior experience, we utilized the “clump_data” function in the “TwoSampleMR” package of RStudio version 4.2.1 to identify SNPs in the gut microbiota dataset with a genetic distance of 250 kb and an r^2^ < 0.01. When obtaining SNPs for BMR, we identified SNPs in the ukb-a-268 dataset with a significance level of *p* < 5e-08, a genetic distance of 10,000 kb, and *r*^2^ < 0.001. When obtaining SNPs for OA, we identified SNPs in the dataset with *p* < 5e-08, a genetic distance of 10,000 kb, and *r*^2^ < 0.001. For secondary datasets with gut microbiota in two-sample MR, the threshold was adjusted to p1 = 5e-06, *r*^2^ = 0.01, and kb = 1,000. This modification was necessary due to a limited number of SNPs.

To address assumption 1, we utilized the PhenoScanner database to identify potential confounding factors, including body mass index and smoking, within the dataset. Then, we harmonized the exposure and outcome datasets by removing palindromic SNPs with allele frequencies close to 0.5. We ensured the strength of the instrumental variables by calculating the F-statistic using the formula: *F* = (n − k − 1)/k × [R^2/(1 − R^2)], where R^2 represents the cumulative explained variance in the selected SNPs, *n* is the sample size, and k is the number of SNPs in the analysis. A value of F greater than 10 indicates that the exposure strength is sufficient to mitigate weak instrumental bias in a two-sample model (Burgess et al., 2011). The selected SNPs for gut microbiota and BMR are presented in Supplementary Tables 1–3.

### 2.4 Statistical analysis

We conducted a bidirectional two-sample MR analysis to assess the relationship between the gut microbiota and OA. The primary analysis utilized the inverse variance-weighted (IVW) method to obtain unbiased estimates of the causal relationship between exposure and outcome. Secondary analyses were conducted using the weighted median method (Bowden et al., 2016) and MR-Egger regression. Cochran’s Q test, based on IVW estimates, was employed to detect heterogeneity among IVs (Pierce and Burgess, 2013). In the presence of heterogeneity, we applied the IVW random-effects model for analysis. We assessed the potential impact of directional pleiotropy by testing the intercept value of the MR-Egger regression. A *p*-value greater than 0.05 indicates weaker evidence for pleiotropic effects in causal analysis (Burgess and Thompson, 2017). Additionally, we utilized the Mendelian Randomization Pleiotropy RESidual Sum and Outlier (MR-PRESSO) method to detect and adjust for horizontal pleiotropy and potential outliers. This approach provides more reliable and robust estimates of causal effects (Gao et al., 2023).

## 3 Results

### 3.1 Causal associations of gut microbiota with OA statistical analysis

**Figure 3:**
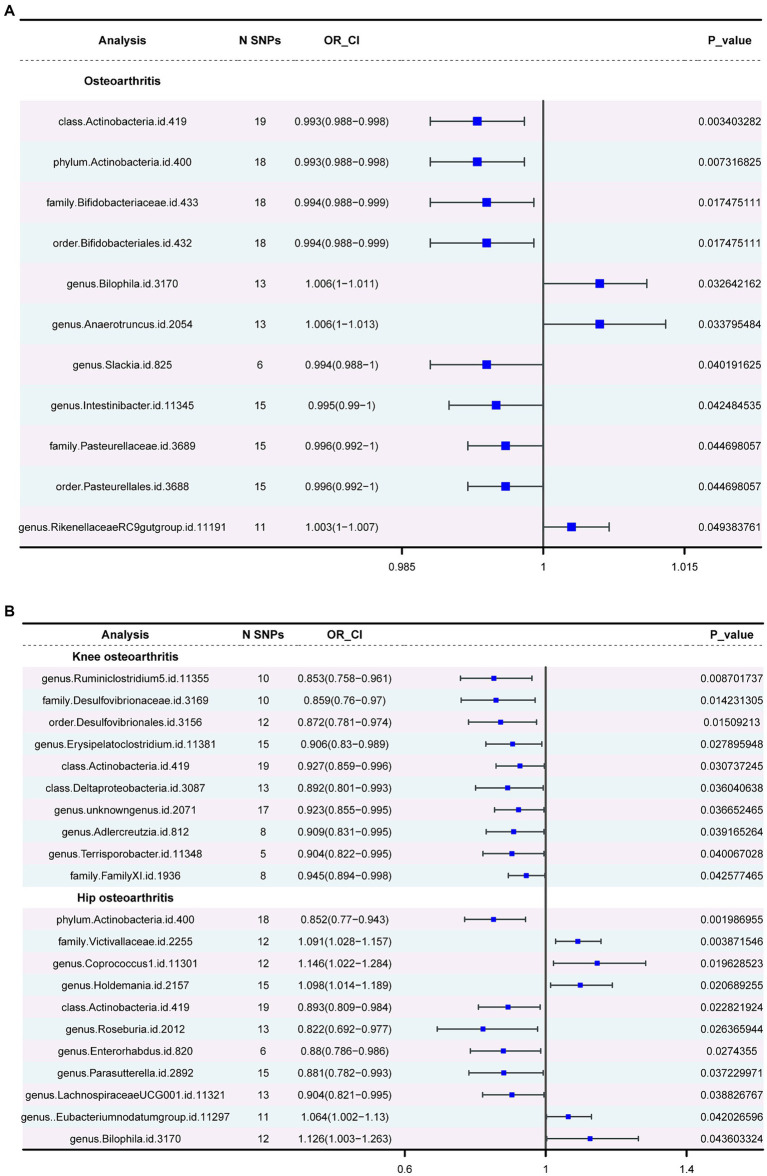
The forest plot of gut microbiota with an inverse variance weight (IVW) < 0.05. **(A)** Odds ratios (OR) for the association between gut microbiota and OA. **(B)**. Odds ratios (OR) for the association between gut microbiota and KOA, HOA.

Three major methods were employed to investigate the association between gut microbiota and OA. The OA dataset was considered the primary outcome, with the KOA and HOA datasets serving as secondary outcomes. In the primary outcome analysis (Supplementary Table 4), negative correlations were observed for class *Actinobacteria*, phylum *Actinobacteria*, family *Bifidobacteriaceae*, *Pasteurellaceae*, order *Bifidobacteriales* and *Pasteurellales*, and genus *Slackia*. Conversely, positive correlations were observed for the genera *Bilophila*, *Anaerotruncus*, and *RikenellaceaeRC9* gut group. In the secondary outcomes, only the class *Actinobacteria* showed consistency with the primary outcome. Additionally, in the HOA dataset, consistency with the primary outcome was observed for the phylum *Actinobacteria* and the genus *Bilophila*. Figure 3 depicts the forest plot of odds ratios (OR) for the association between gut microbiota and OA.

**Figure 4:**
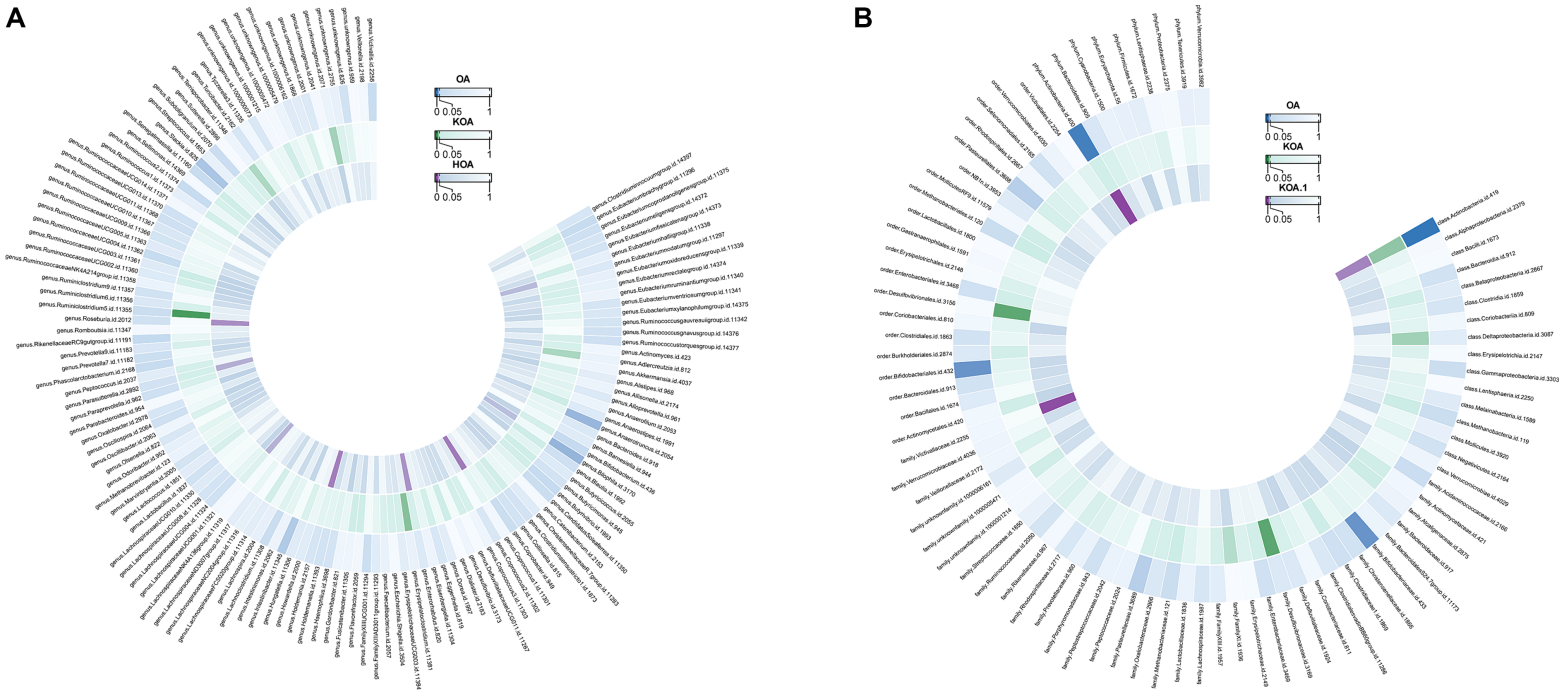
Circular heatmap of complete *p*-value results for 211 gut microbiotas using the IVW method about OA. **(A)**. Causal relationship between 131 gut microbiota genera and OA. **(B)**. Causal relationship between 80 non-microbiota genera and OA.

*Actinobacteria* (OR=0.992886277, *p*-value = 0.003) showed a negative association with the likelihood of OA occurrence. Strong correlations were observed in both KOA and HOA (OR = 0.927237553 and 0.892581219), suggesting a potential link with OA development. Figure 4 displays two circular heatmaps of complete *p*-value results for 211 gut microbiotas using the IVW method about OA.

Reverse analyses were conducted for the taxa showing positive results, which revealed no significant impact of OA on gut microbiota (Supplementary Table 8). To further confirm the robustness of the results, we conducted multiple sensitivity tests (Supplementary Table 8). Moreover, all results from Cochran’s Q test were above 0.05, indicating no significant heterogeneity. Furthermore, neither the MR-Egger intercept test nor the global test *p*-value yielded statistically significant results, indicating the lack of horizontal pleiotropy.

### 3.2 Causal associations of gut microbiota with BMR levels

Similarly, we conducted a two-sample analysis to examine the relationship between gut microbiota and BMR. The IVW fixed-effects analysis in the Supplementary Table 5 reveals a negative correlation between order *Methanobacteriales*, family *Methanobacteriaceae*, class *Methanobacteria*, and BMR levels (*β* = −0.014, *p* = 0.002). Genus *Lachnospiraceae UCG004*, order *Bifidobacteriales*, family *Bifidobacteriaceae*, and Family *XIII*, class *Actinobacteria*, also exhibited negative correlations with BMR levels. Only the genus *Coprobacter* showed a positive correlation with BMR levels, and similar causal estimates were obtained through WM analysis.

A series of sensitivity analyses were performed, including WM, Cochran’s Q test, MR-Egger regression, and intercept test (Supplementary Table 5). Furthermore, all *p*-values from the MR-Egger intercept test were greater than 0.05, indicating no evidence of horizontal pleiotropy. Three positive results indicating heterogeneity were found in Cochran’s Q test. Subsequently, we conducted an IVW random-effects model analysis and utilized it as the primary outcome. Reverse analyses revealed no impact of BMR on the gut microbiota, showing positive associations (Supplementary Table 9).

### 3.3 Causal associations of BMR with OA

Finally, we conducted a two-sample MR analysis to examine the impact of BMR on OA. After removing confounding SNPs related to factors such as obesity and smoking, the resulting SNPs are listed in Supplementary Table 3. Supplementary Table 6 presents the results. IVW fixed-effects analysis shows a positive correlation between BMR levels and OA, with a more significant positive correlation observed in the secondary datasets KOA and HOA. Furthermore, heterogeneity tests and pleiotropy tests strengthened the robustness of the study’s findings.

### 3.4 Mediation effect of BMR levels in the causal association between gut microbiota and OA

Previously, it was believed that energy demands increase during illness, as elevated measurements of BMR are frequently observed during disease states (Gibney, 2000). Therefore, we conducted reverse analyses but found no significant association between OA and BMR (Supplementary Table 7).

Summarizing the results, as shown in Table 2, positive results were found for the class *Actinobacteria* in both the primary and secondary datasets. BMR plays a significant role in the causal pathway from class *Actinobacteria* to OA, with a mediated effect of 2.59%. The effect is most pronounced in KOA, with a mediated effect of 16.40%, and a 5.15% mediated effect in HOA. It also exerts a 3.98% mediated effect in the pathway from order *Bifidobacteriales* and family *Bifidobacteriaceae* to OA.

**Table 2:** Two-step Mendelian randomization.

Exposure	Mediation	Total effect	(Beta) A	(Beta) B	Indirect effect (Beta)	Mediation effect/Total effect
Osteoarthritis						
class.Actinobacteria.id.419	BMR	−0.007139	−0.0219	0.00845	−0.000185211	0.025942973
order.Bifidobacteriales.id.432	BMR	−0.006381	−0.0301	0.00845	−0.000254304	0.039854581
family.Bifidobacteriaceae.id.433	BMR	−0.006381	−0.0301	0.00845	−0.000254304	0.039854581
Knee osteoarthritis						
class.Actinobacteria.id.419	BMR	−0.075545	−0.0219	0.56506	−0.012390196	0.164009749
Hip osteoarthritis						
class.Actinobacteria.id.419	BMR	−0.113638	−0.0219	0.26678	−0.00584971	0.051476808

## 4 Discussion

This study utilized large-scale GWAS summary data and employed a two-sample, two-step bidirectional MR analysis to investigate the associations between gut microbiota, BMR levels, and OA. This study represents the first comprehensive and in-depth investigation into the causal relationships between BMR-mediated gut microbiota and OA based on publicly available GWAS data. According to our extensively corrected study results, an increase in the abundance of *Actinobacteria* and *Bifidobacteria* may negatively correlate with the risk of developing OA. Additionally, we observed that the decrease in OA risk may be mediated by a reduction in BMR (mediated proportions = 2.59 and 3.98%, respectively). Consistent directional effects were observed in all analyses using MR Egger and the weighted median method, suggesting that targeting *Bifidobacteria* and *Actinobacteria* may hold promise for preventing OA.

The human gastrointestinal tract is home to trillions of symbiotic bacteria, forming a mutually beneficial relationship with the human body and contributing to the maintenance of our health (Qin et al., 2010). Recent research has highlighted the crucial role of gut microbiota imbalance in metabolic diseases (Zhang et al., 2015; Stewart et al., 2016). OA is classified as a metabolic disease (Gowd et al., 2020), and there are often interconnections between different metabolic disorders. For example, obesity is a risk factor for type 2 diabetes, and excessive weight can also contribute to the onset of non-alcoholic fatty liver disease (Wu et al., 2019). Adverse changes in gut microbiota can contribute to the beginning of metabolic syndrome and inflammation, both playing crucial roles in the development and advancement of OA (Jin et al., 2015). Recent findings indicate that the gut microbiota may stimulate systemic inflammation by triggering the innate immune response. This discovery establishes a potential link between metabolism and the establishment of mechanisms associated with OA (Metcalfe et al., 2012).

Previous experimental studies have suggested that prebiotic oligosaccharides derived from corn starch and lentil diets can promote the growth of *Bifidobacteria* while reducing the abundance of Firmicutes bacteria. These bacteria could benefit overweight and obese populations (Barczynska et al., 2016; Siva et al., 2018; Panichsillaphakit et al., 2021). It may even reduce the occurrence of OA. Eric M. Schott’s et al. animal experiment also indicated that lean mice predominantly harbor *Bacteroidetes* and *Actinobacteria*, with a notably abundant presence of the genus *Bifidobacterium* (Schott et al., 2018). Research on *Bifidobacterium* and OA is extensive, indicating that *Bifidobacterium* may influence OA through its metabolites, specifically short-chain fatty acids, including acetate, propionate, and butyrate. Butyrate, for instance, has been discovered to boost osteoblast function and suppress osteoclast activity, consequently fostering comprehensive bone health (Medawar et al., 2021). Butyrate induces autophagy and reverses detrimental autophagic processes, thereby ameliorating the inflammatory milieu, reducing necroptotic tendencies, and potentially improving osteoarthritis progression through modulation of intestinal environment and autophagic flux (Cho et al., 2022).This mechanism is pivotal in regulating the immune system and maintaining metabolic balance (Wang et al., 2015). Schott et al. (2018) demonstrated that saturated fatty acids are toxic to OA pathogenesis (Csekar et al., 2017). Supplementation with prebiotic fibers can reverse the detrimental effects of a fat-rich diet (especially saturated fatty acids) on the gut microbiota by increasing the abundance of *Bifidobacterium*. *Bifidobacterium* also contributes to absorbing and utilizing essential nutrients, including calcium, vitamin D, iron, and phosphorus. These nutrients are necessary for muscle metabolism (Montazeri-Najafabady et al., 2019).

Moreover, *Bifidobacterium* exhibits a richness in genes encoding proteins associated with carbohydrate and amino acid transport and metabolism, which influence protein synthesis and the utilization of nutrients (Tu et al., 2023). This could represent one of the potential mechanisms by which *Bifidobacterium* may mitigate the onset of OA. Henrotin et al. (2021) demonstrated that oral administration of *Bifidobacterium longum CBi0703* for 12 weeks can reduce cartilage structural damage and decrease the degradation of type II collagen, suggesting a potential preventive role in the development of OA.

Our MR analysis observed significant negative associations between *Bifidobacteria* and *Actinobacteria* with BMR. Additionally, *Methanobacteriales* also displayed notable differences in association with BMR. Various factors, including temperature, hormone levels, body surface area, muscle mass, gender, genetic factors, age, and nutritional status, influence BMR. BMR signifies the daily energy needs essential for sustaining fundamental bodily functions. It significantly contributes to energy expenditure (Shetty, 2005) and is an essential parameter for estimating daily energy needs (Ferro-Luzzi, 2005). Gut microbiota plays a role in regulating metabolism and maintaining energy balance (Nieuwdorp et al., 2014).

On the one hand, the human body metabolizes nutrients from food for energy metabolism (Li et al., 2023), and gut microbiota regulates gastrointestinal functions, influencing the digestion, absorption, and breakdown of food, thus affecting BMR. On the other hand, muscle metabolism is a critical factor in determining the basal metabolic rate, and a positive correlation has been observed between the skeletal muscle mass index and BMR (*β* = 30.96, *p* < 0.01) (Kim et al., 2016). The “gut-bone/muscle” axis has become a focal point of interest within the realms of bone health and orthopedic diseases. Numerous studies suggest that the composition of gut microbiota is involved in the pathogenesis of orthopedic diseases through various pathways (Tu et al., 2021; Zhang et al., 2021). Nardone et al. (2021) found that the gut-muscle axis plays a significant role in treating muscle wasting. They hypothesized that targeted or supplemental treatment of the gut microbiota could be implemented for patients with muscle wasting. However, limited literature discusses the specific regulatory mechanisms of the gut microbiota on BMR.

In recent decades, studies on BMR have predominantly focused on metabolism-related disorders and obesity, with successful applications found in the treatment of diabetes (Guan et al., 2022). It is well known that risk factors for OA include aging, diet, and obesity, all of which are associated with the body’s metabolism (Mooney et al., 2011). Traditionally, it has been understood that obesity or aging often leads to a decrease in the body’s metabolic rate. However, in our study, we observed a notable positive correlation between BMR and the occurrence of OA.

The consensus on whether BMR directly influences OA has not been reached in prior retrospective studies. Mobasheri et al. (2017) pointed out that OA is a metabolic disorder, emphasizing the crucial role of metabolism in the functionality of cartilage and synovial joints. They provided a comprehensive overview of the involvement of metabolism in the pathogenesis of OA. Several biological mechanisms can elucidate the positive correlation between elevated BMR and the risk of OA. Primarily, the theory of ‘free radicals’ proposes that the oxidative stress produced through metabolism is harmful, with the cumulative damage compromising bodily systems over time (Park and Yeo, 2013). Secondly, a higher BMR implies a greater cellular energy demand to meet metabolic requirements (Wu et al., 2022). Mitochondria function as the primary hub of cellular metabolism, and the mitochondrial respiratory chain significantly contributes to generating cellular reactive oxygen species (ROS). While ROS plays a crucial role in physiological signaling, elevated ROS poses significant pathological risks as a medium for the progression of OA (Chen et al., 2008). Additionally, research suggests a connection between metabolism and chronic inflammation (Lepetsos et al., 2019). Under the premise of a high BMR, inflammation and the degradation of protein biosynthesis increase, inducing catabolic metabolism and advancing the progression of OA (Hotamisligil, 2006). The production of excess metabolites and nutrients further promotes the advancement of inflammation (Wang et al., 2015). Inflammation, by affecting chondrocyte differentiation, expression of metalloproteinases, and the formation of aggregates, ultimately results in cartilage degradation and joint damage, influencing the onset of OA (Robinson et al., 2016).

In summary, our discoveries propose that BMR may function not only as a metabolic indicator but also contribute to mediating the pathogenic mechanisms of OA. These results potentially establish the involvement of specific gut microbiota in systemic inflammation and metabolic responses. Our investigation has unveiled intricate interactions and potential mechanisms that underlie the relationship between gut microbiota, BMR levels, and OA. Key mechanisms may involve inflammation or immune imbalance triggered by dysbiosis of the gut microbiota and metabolic alterations induced by the “gut-bone/muscle” axis. Subsequent research endeavors should delve deeper into potential therapeutic strategies for early intervention in osteoarthritis by modulating metabolism through the regulation of specific microbiota, such as *Bifidobacteria* and *Actinobacteria*.

However, it is crucial to recognize the limitations of this study. Firstly, MR hinges on the fundamental assumption that the genetic variations acting as instrumental variables influence the outcome exclusively through the exposure factor. Although we endeavored to mitigate confounding factors and applied diverse methods to address pleiotropy, the presence of unmeasurable confounding factors remains a possibility. Secondly, our study predominantly concentrates on the European population, and the applicability of our findings to other populations might be constrained. Our next plan is to select datasets with larger sample sizes and more racial sources for analysis to obtain more robust results. We acknowledge that, in addition to BMR levels, there may be other potential mediators that might contribute to the observed relationship between gut microbiota and OA. Subsequent investigations could delve into these mediating factors to acquire a more thorough understanding of the intricate interactions at play. Furthermore, if more comprehensive gut microbiota data become available in the future, it would be a valuable supplement to our current findings.

## 5 Conclusion

In conclusion, our bidirectional Mendelian randomization analysis indicates a causal relationship between specific gut microbiota and a reduced risk of OA, while the hypothesis of reverse causation does not hold. Significantly, our findings indicate that BMR plays a role in mediating the influence of *Actinobacteria* and *Bifidobacteria* on OA. To enhance comprehension of the established correlation between gut microbiota and OA, future studies should prioritize investigating potential mechanistic pathways and accounting for potential confounding factors such as diet, lifestyle, and medications when presenting these findings. Further experimental and clinical studies are needed to validate and expand upon our findings. We aspire that our study acts as a catalyst for further exploration in this field, making a meaningful contribution to the ongoing endeavors aimed at addressing the potential risks associated with OA.

## Data Availability

The original contributions presented in the study are included in the article/Supplementary material, further inquiries can be directed to the corresponding authors.

## Footnotes

^1^ https://gwas.mrcieu.ac.uk/

^2^ http://gwas-api.mrcieu.ac.uk/
