# Corrigendum: Basal metabolic rate mediates the causal relationship between gut microbiota and osteoarthritis: a two-step bidirectional Mendelian randomization study

**DOI:** 10.3389/fmicb.2024.1510272

**Published:** 2024-10-29

**Authors:** Jiachen Li, Jianhui Liang, Yang Liu, Weichao Sun, Wei Sun

**Affiliations:** ^1^Department of Orthopedics, Shenzhen Second People's Hospital/First Affiliated Hospital of Shenzhen University, Shenzhen, Guangdong, China; ^2^Shantou University Medical College, Shantou, China; ^3^The Central Laboratory, Shenzhen Second People's Hospital/First Affiliated Hospital of Shenzhen University, Shenzhen, Guangdong, China

**Keywords:** basal metabolic rate, gut microbiota, mediation, Mendelian randomization, osteoarthritis

In the published article, there was an error in the affiliation for Wei Sun. Instead of “^2^Shantou University Medical College, Shantou, China”, it should be “^1^Department of Orthopedics, Shenzhen Second People's Hospital/First Affiliated Hospital of Shenzhen University, Shenzhen, Guangdong, China”.

In the published article, there was also an error in the Funding statement. The funding information for Guangdong Provincial High-Level Clinical Key Specialty-Orthopedics was mistakenly omitted. The corrected Funding statement appears below.

